# Significant predictors of medically diagnosed chronic obstructive pulmonary disease in patients with preserved ratio impaired spirometry: a 3-year cohort study

**DOI:** 10.1186/s12931-018-0896-7

**Published:** 2018-09-24

**Authors:** Hye Jung Park, Min Kwang Byun, Chin Kook Rhee, Kyungjoo Kim, Hyung Jung Kim, Kwang-Ha Yoo

**Affiliations:** 1 Department of Internal Medicine, Gangnam Severance Hospital, Yonsei University College of Medicine, 211 Eonju-ro Gangnam-gu, Seoul, 06273 Korea; 2 Division of Pulmonary, Allergy and Critical Care Medicine, Department of Internal Medicine, Seoul St Mary’s Hospital, College of Medicine, The Catholic University of Korea, Seoul, Korea; 3 Department of Internal Medicine, Konkuk University School of Medicine, Seoul, Korea

**Keywords:** Chronic obstructive pulmonary disorder, Prognosis, Spirometry

## Abstract

**Background:**

Preserved ratio impaired spirometry (PRISm) is an incompletely understood respiratory condition. We investigated the incidence and significant predictive factors of chronic obstructive pulmonary disease (COPD) in PRISm patients.

**Methods:**

From 11,922 subjects registered in the Korea National Health and Nutrition Examination Survey, never or light smokers, young subjects, and those already medically diagnosed with COPD (defined by ICD-10 code and prescribed medication) were excluded. The 2666 remaining subjects were categorized into PRISm (normal forced expiratory volume in the first second [FEV_1_]/force vital capacity [FVC] [≥ 0.7] and low FEV_1_ (< 80%); *n* = 313); normal (*n* = 1666); and unrevealed COPD groups (FEV_1_/FVC ratio <  0.7; *n* = 687). These groups were compared using matched Health Insurance Review and Assessment Service data over a 3-year follow-up.

**Results:**

COPD incidence in PRISm patients (17/1000 person-year [PY]) was higher than that in normal subjects (4.3/1000 PY; *P* <  0.001), but lower than that in unrevealed COPD patients (45/1000 PY; *P* < 0.001). PRISm patients visited hospitals, took COPD medication, and incurred hospitalization costs more frequently than normal subjects, but less frequently than unrevealed COPD patients. In the overall sample, age, FVC, FEV_1_, dyspnea, and wheezing were significant predictors of COPD, but in PRISm patients, only age (OR, 1.14; *P* = 0.002) and wheezing (OR, 4.56; *P* = 0.04) were significant predictors.

**Conclusion:**

PRISm patients are likely to develop COPD, and should be monitored carefully, especially older patients and those with wheezing, regardless of lung function.

## Background

Despite the escalating prevalence and economic burden of chronic obstructive pulmonary disease (COPD), many COPD cases remain undiagnosed worldwide [1–3]. A lack of awareness of COPD, lack of educational programs concerning COPD, poor physician adherence to guidelines, and low usage of pulmonary function tests leads to underdiagnosis of COPD [4, 5]. Many studies have reported that patients with early COPD or even pre-COPD (e.g., smokers or subjects with impaired lung function) have respiratory symptoms and utilize medical support [6, 7]. This has emphasized early-diagnosis and early-treatment of COPD. However, subjects with preserved ratio impaired spirometry (PRISm) are often missed. PRISm patients do not meet COPD criteria [8], with a preserved ratio of force expiratory volume in the first second [FEV_1_]/forced vital capacity [FVC] (> 0.7), but have reduced FEV_1_ (< 80%, predicted), yet exhibit increased respiratory symptoms, decreased activity, increased comorbidity, and increased mortality [7, 9–14]. Wan et al. described PRISm as a COPD subtype with increased emphysema and gas trapping [15]. Lung density on computed tomography is significantly associated with lung function in PRISm [16]. Thus, some aspects of PRISm are associated with COPD development with worsening of lung function; but the COPD incidence in PRISm patients has rarely been reported.

Tobacco smoking, ageing, air pollution, poor nutritional status, impaired lung function, and underlying asthma are established risk factors for COPD [17, 18]. However, the risk factors associated with COPD in PRISm remain unknown. We sought to elucidate the incidence of COPD in PRISm patients and to identify the significant risk factors for COPD in PRISm, using Korean national cohort data.

## Methods

### Subjects and study design

**Fig. 1:**
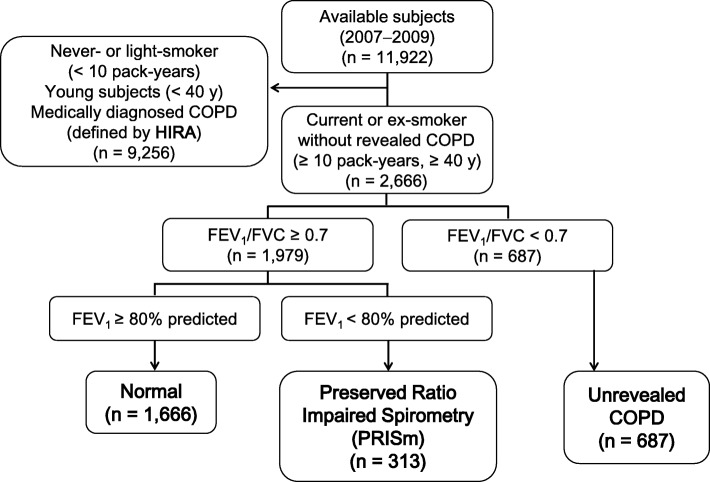
Subject selection and group assignment based on the KNHANES and HIRA data. KNHANES, Korea National Health and Nutrition Examination Survey; HIRA, Health Insurance Review & Assessment; COPD, chronic obstructive pulmonary disease; FEV_1_, forced expiratory volume for 1 s; FVC, forced vital capacity

We used the cross-sectional the Korea National Health and Nutrition Examination Survey (KNHANES) data of 2007–2009 and KNHANES-matched Health Insurance Review and Assessment (HIRA) cohort data of 2006–2012. A total of 11,922 subjects were available in KNHANES. Among them, never- or light-smokers (< 10 pack-years), young subjects (< 40 years), and patients already medically diagnosed with COPD (based on the ICD-10 code and prescribed medication in HIRA), were excluded (*n* = 9256). We categorized the remaining 2666 subjects into 3 groups based on spirometry (Fig. 1). The normal group (*n* = 1666) had a normal FEV_1_/FVC ratio (≥ 0.7) and normal spirometry (FEV_1_ ≥ 80% predicted). PRISm subjects (*n* = 313) had a normal FEV_1_/FVC ratio (≥ 0.7) and decreased lung function (FEV_1_ < 80% predicted). Unrevealed COPD subjects had a decreased FEV_1_/FVC ratio (< 0.7), regardless of FEV_1_ and FVC. KNHANES data did not include post-bronchodilator FEV_1_ and FVC, which are recommended in the guidelines [8, 19]; we therefore used pre-bronchodilator FEV_1_ and FVC values.

### KNHANES and HIRA data

KNHANES data were derived from a national large-scale cross-sectional survey conducted by the Korean government, via the Korea Centers for Disease Control and Prevention. This data were obtained from a well-designed national program with complex, multistage probability sample extraction to reflect the total population of Korea. KNHANES data include age, sex, height, weight, self-reported smoking history, self-reported co-morbidity (answers to the following questions: do you have [the disease, e.g., asthma] diagnosed by a doctor?), results of spirometry tests obtained using Korean classic guidelines [20], and self-reported respiratory symptoms (answers to the following questions: do you have [symptom, e.g., cough for 3 months]?). We enrolled subjects based on age, smoking history (in pack-years, PY), and lung function in the KNHANES data. Other baseline characteristics were also obtained from the KNHANES data.

Subjects enrolled in the KNHANES database have KNHANES-matched HIRA data. HIRA data were obtained from claims from the national health insurance system, which uniquely covers virtually all residents in Korea. It contains the diagnostic code, medical utilization (including hospital admission history and prescribed medication), and costs for several years [21].

### Parameter definition

Contrary to the established spirometry-based diagnostic criterion for COPD (FEV_1_/FVC < 0.7), “medically diagnosed COPD” was defined by diagnostic code and prescribed medication [22, 23]. Medically diagnosed COPD patients met all of the following criteria: 1) age ≥ 40 years; 2) ICD-10 codes for COPD or emphysema (J43.0×-J44.x, with the exception of J43.0 as primary or secondary [within fourth position] diagnosis); and 3) the use of more than 1 of the following COPD medications at least twice per year: long-acting muscarinic antagonist, long-acting beta-2 agonist (LABA), fixed-dose inhaled corticosteroid with LABA, short-acting muscarinic antagonist (SAMA), short-acting beta-2 agonist (SABA), SAMA with SABA, phosphodiesterase-4 (PDE-4) inhibitor, systemic beta agonist, or methylxanthine.

Hospitalization cost was defined as any medical utilization costs for inpatient services, confined to admissions with an ICD-10 code for COPD (J43.x–J44.x, except J430) or COPD-related diseases (pneumonia: J12.x–J17.x; pulmonary thromboembolism: I26, I26.0, and I26.9; dyspnea: R06.0; or acute respiratory distress syndrome: J80). Costs were presented in US dollar (USD), using an exchange rate of 1 USD = 1090 Korean Won (exchange rate as on February 9, 2018).

Chronic bronchitis was defined as self-reported chronic cough or sputum persisting for at least 3 months, in at least 2 consecutive years.

### Outcomes

**Fig. 2:**
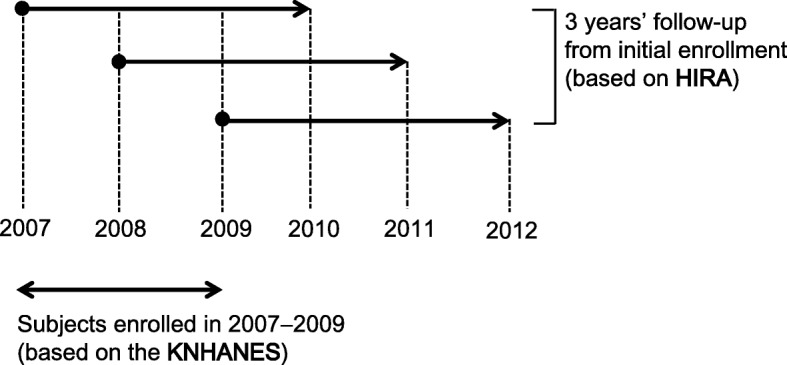
Scheme of study and summary of data presentation. KNHANES, Korea National Health and Nutrition Examination Survey; HIRA, Health Insurance Review & Assessment

We analyzed the 3-year follow-up outcomes from HIRA data (Fig. 2). The incidence of medically diagnosed COPD was the primary outcome. Hospital visits, number and type of prescribed medication, and hospitalization cost were secondary outcomes. Furthermore, we sought to identify significant factors that predicted a COPD diagnosis by group.

### Ethics

This study was approved by the Institutional Review Board of Gangnam Severance Hospital (number: 3–2017-0395). The requirement for obtaining informed consent from the patients was waived due to the retrospective nature of this study.

### Statistical analyses

We compared the baseline characteristics, COPD incidence, hospital visits, medication use, and hospitalization cost between groups using *χ*^2^ tests (categorized variables) and analysis of variance with Bonferroni post-hoc test (continuous variables). Univariate and multivariate logistic regression analyses were conducted to identify factors that predicted COPD diagnosis. In multivariate analysis, only factors found significant in univariate analysis were included as co-variables. FEV_1_/FVC was not used in multivariate analysis, because of increased multicollinearity (variance inflation factor = 23.81). *P* < 0.05 was considered to indicate statistical significance.

## Results

### Demographics of subjects by group

Unrevealed COPD subjects (64.48 ± 9.54 years) were significantly older than subjects in the normal (54.57 ± 10.52 years; *P* < 0.001) and PRISm (55.97 ± 10.85 years; *P* < 0.001) groups. Most subjects were men, and the sex distribution was similar among groups. Height and weight were less in the unrevealed COPD than in the normal and PRISm subjects. Smoking PY was heavier in the unrevealed COPD group than in the normal and PRISm groups. However, PRISm subjects were more often current-smokers (61.7%) than were normal (51.5%; *P* = 0.003) and unrevealed COPD (53.4%; *P* = 0.045) subjects. Hyperlipidemia was less common in the unrevealed COPD (6.8%) than in the normal group (10.0%; *P* = 0.048). Acute coronary syndrome was more common in the unrevealed COPD (2.3%) than in the normal (1.0%; *P* = 0.042) group. Diabetes mellitus was significantly more prevalent in PRISm (20.1%) than in normal (10.4%; *P* < 0.001) and unrevealed COPD (12.2%; *P* = 0.003) subjects. Pulmonary tuberculosis and asthma was particularly prevalent in the unrevealed COPD group (Table 1).

**Table 1:** Demographics of subjects according to the group

	Normal	PRISm	Unrevealed COPD	*P*-value	*P-*value*	*P-*value+	*P-*value‡
Age	54.57 ± 10.52	55.97 ± 10.85	64.48 ± 9.54	< 0.001	0.083	< 0.001	< 0.001
Male, *n* (%)	1560 (93.6)	286 (91.4)	654 (95.2)	0.063	0.426	0.432	0.054
Height (cm)	167.14 ± 6.94	166.79 ± 6.94	165.97 ± 6.56	< 0.001	0.998	<.0.001	0.236
Weight (kg)	68.29 ± 9.91	68.66 ± 11.6	63.7 ± 9.8	< 0.001	0.998	< 0.001	< 0.001
Smoking history							
Current smoking, *n* (%)	858 (51.5)	193 (61.7)	367 (53.4)	0.004	0.003	0.999	0.045
Pack-years	28.62 ± 17.11	33.20 ± 20.34	36.58 ± 21.14	< 0.001	< 0.001	< 0.001	0.026
Co-morbidity, *n* (%)							
Hypertension	453 (27.2)	91 (29.1)	209 (30.4)	0.269	0.998	0.336	0.999
Hyperlipidemia	166 (10.0)	34 (10.9)	47 (6.8)	0.035	0.998	0.048	0.092
Stroke	48 (2.9)	14 (4.5)	18 (2.6)	0.252	0.414	0.999	0.368
Acute coronary syndrome	17 (1.0)	8 (2.6)	16 (2.3)	0.019	0.077	0.042	0.999
Diabetes mellitus	174 (10.4)	63 (20.1)	84 (12.2)	< 0.001	< 0.001	0.624	0.003
Pulmonary tuberculosis	124 (7.4)	21 (6.7)	109 (15.9)	< 0.001	0.999	< 0.001	< 0.001
Asthma	20 (1.2)	15 (4.8)	65 (9.5)	< 0.001	< 0.001	< 0.001	0.024
Lung function test							
FVC % predicted	92.96 ± 10.02	72.55 ± 9.45	88.51 ± 15.02	< 0.001	< 0.001	< 0.001	< 0.001
FEV_1_% predicted	94.66 ± 9.14	72.8 ± 6.72	74.18 ± 16.57	< 0.001	< 0.001	< 0.001	0.035
FEV_1_/FVC	0.79 ± 0.05	0.77 ± 0.06	0.61 ± 0.09	< 0.001	< 0.001	< 0.001	0.006
Respiratory symptoms, *n* (%)							
Cough for more than 3 months	1 (0.1)	2 (0.6)	19 (2.8)	< 0.001	0.047	< 0.001	0.091
Sputum for more than 3 months	4 (0.2)	2 (0.6)	18 (2.6)	< 0.001	0.999	< 0.001	0.104
Dyspnea	10 (0.6)	3 (1.0)	31 (4.5)	< 0.001	0.999	< 0.001	0.012
Wheezing	116 (7.0)	37 (11.8)	154 (22.4)	< 0.001	0.009	< 0.001	< 0.001
Chronic bronchitis	4 (0.2)	2 (0.6)	21 (3.1)	< 0.001	0.717	< 0.001	0.054
Total	1666	313	687				

Data are presented as mean ± standard deviation or number (percentage)

* *P*-value for comparison between normal and PRISm group; + *P*-value for comparison between normal and unrevealed COPD group; ‡ *P*-value for comparison between PRISm and unrevealed COPD group

*PRISm* preserved ratio impaired spirometry, *COPD* chronic obstructive pulmonary disease, *FEV*_*1*_ forced expiratory volume for 1 s, *FVC* forced vital capacity

FVC was significantly lower in the PRISm (72.55 ± 9.45%) than in the normal (92.96 ± 10.02%; *P* < 0.001) and unrevealed COPD (88.51 ± 15.02%; *P* < 0.001) groups. FEV_1_ followed a similar pattern. However, the FEV_1_/FVC ratio was significantly lower in the unrevealed COPD (0.61 ± 0.09) than in the normal (0.79 ± 0.05, *P* < 0.001) and PRISm (0.77 ± 0.06, *P* = 0.035) groups. Wheezing was more prevalent in PRISm (11.8%) patients than in normal subjects (7.0%; *P* = 0.009), but less prevalent than in the unrevealed COPD group (22.4%, *P* < 0.001). Other respiratory symptoms followed a similar pattern (Table 1).

### COPD incidence, medication and hospital utilization, and cost

The COPD incidence in PRISm subjects (17.0/1000 person year [PY]) was significantly higher than that in normal subjects (4.4/1000 PY; *P* < 0.001); however, that in unrevealed COPD individuals (45.1/1000 PY) was significantly higher than that in PRISm individuals (*P* < 0.001). The PRISm group (13.1%) significantly more often visited the hospital than the normal group (7.3%; *P* = 0.002), but less often than the unrevealed COPD group (24.6%; *P* < 0.001). The type and number of prescribed medications followed a similar pattern. Hospitalization cost in the PRISm group (398.61 ± 1975.51 USD) was almost double that in the normal group (186.17 ± 1411.24 USD; *P* = 0.297); however, that in the unrevealed COPD group (750.71 ± 3216.02 USD; *P* = 0.041) was larger than that in the PRISm group (Table 2).

**Table 2:** COPD incidence, medication and hospital utilization, and cost

	Normal	PRISm	Unrevealed COPD	*P*-value	*P-*value*	*P-*value+	*P-*value‡
COPD incidence (/1000PY)	4.4	17.0	45.1	< 0.001	< 0.001	< 0.001	< 0.001
OPD visit, *n* (%)	51 (3.1)	22 (7.0)	131 (19.1)	< 0.001	0.002	< 0.001	< 0.001
No. of OPD visit	0.10 ± 0.91	0.48 ± 2.96	1.86 ± 6.37	< 0.001	0.243	< 0.001	< 0.001
Hospitalization, *n* (%)	79 (4.7)	29 (9.3)	83 (12.1)	< 0.001	0.004	< 0.001	0.571
ER visit, n (%)	23 (1.4)	12 (3.8)	36 (5.2)	< 0.001	0.008	< 0.001	0.999
ICU admission, n (%)	12 (0.7)	6 (1.9)	19 (2.8)	< 0.001	0.122	< 0.001	0.999
Total hospital visit, n (%)	121 (7.3)	41 (13.1)	169 (24.6)	< 0.001	0.002	< 0.001	< 0.001
ICS, n (%)	4 (0.2)	5 (1.6)	20 (2.9)	< 0.001	0.003	< 0.001	0.651
ICS + LABA, n (%)	2 (0.1)	11 (3.5)	50 (7.3)	< 0.001	< 0.001	< 0.001	0.063
LAMA, n (%)	–	4 (1.3)	44 (6.4)	–	–	–	< 0.001
SAMA, n (%)	12 (0.7)	12 (3.8)	36 (5.2)	< 0.001	< 0.001	< 0.001	0.999
SABA, n (%)	14 (0.8)	11 (3.5)	54 (7.9)	< 0.001	< 0.001	< 0.001	0.029
Systemic bronchodilator, n (%)	28 (1.7)	11 (3.5)	72 (10.5)	< 0.001	0.094	< 0.001	< 0.001
Methylxanthine, n (%)	33 (2.0)	17 (5.4)	101 (14.7)	< 0.001	0.001	< 0.001	< 0.001
Total prescribed medication, n (%)	57 (3.4)	26 (8.3)	127 (18.5)	< 0.001	< 0.001	< 0.001	< 0.001
Hospitalization medical Cost (for 3 years) (USD)	186.17 ± 1411.24	398.61 ± 1975.51	750.71 ± 3216.02	< 0.001	0.297	< 0.001	0.041

Data are presented as mean ± standard deviation or number (percentage)

* *P*-value for comparison between the normal and PRISm group; + *P*-value for comparison between normal and unrevealed COPD group; ‡ *P*-value for comparison between PRISm and unrevealed COPD group

*PRISm* preserved ratio impaired spirometry, *COPD* chronic obstructive pulmonary disease, *PY* person-year, *OPD* outpatient department, *ER* emergency room, *ICU* intensive care unit, *ICS* inhaled corticosteroid, *LABA* long-acting beta-2 agonist, *LAMA* long-acting muscarine antagonist, *SAMA* short-acting muscarine antagonist, *SABA* short-acting beta-2 agonist

### Comparison of baseline characteristics, medical utilization, and costs between subjects with and without medically diagnosed COPD

Among the 2666 subjects, 131 patients (4.9%) were medically diagnosed with COPD during the 3 years’ follow-up. Subjects with medically diagnosed COPD were older and shorter, weighed less, had a heavier smoking history, and more often had a history of pulmonary tuberculosis and asthma than the remaining patients. Although data are not shown, other co-morbidity was not significantly different between groups. Subjects with medically diagnosed COPD had more markedly impaired lung function and severe symptoms than subjects without medically diagnosed COPD. They also more frequently visited hospitals, more frequently used COPD medication, and had greater hospitalization cost than subjects without medically diagnosed COPD (Table 3).

**Table 3:** Comparison of baseline characteristics, medical utilization, and costs between subjects with and without medically diagnosed COPD

	Subjects with medically diagnosed COPD	Subjects without medically diagnosed COPD	*P*-value
Age	68.58 ± 7.77	56.70 ± 11.00	< 0.001
Male, n (%)	123 (93.9)	2377 (93.8)	0.954
Height (cm)	164.04 ± 6.25	166.94 ± 6.86	< 0.001
Weight (kg)	60.35 ± 9.89	67.5 ± 10.2	< 0.001
Smoking history			
Current smoking, n (%)	67 (51.2)	1351 (53.3)	0.631
Pack-years	41.1 ± 23.69	30.7 ± 18.52	< 0.001
Co-morbidity, n (%)			
Pulmonary tuberculosis	28 (21.4)	226 (8.9)	< 0.001
Asthma	3 (26.0)	66 (2.6)	< 0.001
Lung function test			
FVC % predicted	81.14 ± 15.77	89.85 ± 12.85	< 0.001
FEV_1_% predicted	66.37 ± 19.36	87.87 ± 14.17	< 0.001
FEV_1_/FVC	0.59 ± 0.16	0.75 ± 0.09	< 0.001
Respiratory symptoms, n (%)			
Cough for more than 3 months	14 (10.7)	8 (0.3)	< 0.001
Sputum for more than 3 months	11 (8.4)	13 (0.5)	< 0.001
Dyspnea	23 (17.6)	21 (0.8)	< 0.001
Wheezing	60 (45.8)	247 (9.7)	< 0.001
Chronic bronchitis	14 (10.7)	13 (0.5)	< 0.001
OPD visit, n (%)	116 (88.6)	88 (3.5)	< 0.001
No. of OPD visit	10.88 ± 11.77	0.07 ± 0.59	< 0.001
Hospitalization, n (%)	67 (51.2)	124 (4.9)	< 0.001
ER visit, n (%)	35 (26.7)	36 (1.4)	< 0.001
ICU admission, n (%)	17 (13.0)	20 (0.8)	< 0.001
Total hospital visit, n (%)	131 (100)	200 (7.9)	
ICS, n (%)	25 (19.1)	4 (0.2)	< 0.001
ICS + LABA, n (%)	54 (41.2)	9 (0.4)	< 0.001
LAMA, n (%)	42 (32.1)	6 (0.2)	< 0.001
SAMA, n (%)	44 (33.6)	16 (0.6)	< 0.001
SABA, n (%)	60 (45.8)	19 (0.8)	< 0.001
Systemic bronchodilator, n (%)	75 (57.3)	36 (1.4)	< 0.001
Methylxanthine, n (%)	110 (84.0)	41 (1.6)	< 0.001
Total prescribed medication, n (%)	131 (100.0)	79 (3.1)	–
Hospitalization medical Cost (for 3 years) (USD)	4041.23 ± 6633.39	166.17 ± 1286.46	< 0.001
Total	131	2535	

Data are presented as mean ± standard deviation or number (percentage)

*COPD* chronic obstructive pulmonary disease, *FEV*_*1*_ forced expiratory volume for 1 s, *FVC* forced vital capacity, *OPD* outpatient department, *ER* emergency room, *ICU* intensive care unit, *ICS* inhaled corticosteroid, *LABA* long-acting beta-2 agonist, *LAMA* long-acting muscarine antagonist, *SAMA* short-acting muscarine antagonist, *SABA* short-acting beta-2 agonist

### Significant factors for COPD diagnosis in subjects overall

Multivariate analysis of all subjects showed that the possibility of COPD diagnosis was increased to 10.0% with every year’s increase in age (odds ratio [OR], 1.10; 95% confidence interval [CI], 1.07–1.13; *P* < 0.001). A 1% increase in FVC and FEV_1_ was significantly associated with a 3% increase and 5% decrease in COPD diagnosis, respectively (FVC [OR, 1.03; 95% CI, 1.01–1.05; *P* = 0.006] and predicted FEV_1_ [OR, 0.95; 95% CI, 0.93–0.96; *P* < 0.001]). Dyspnea (OR, 3.73; 95% CI, 1.23–7.68; *P* = 0.017), and wheezing (OR, 2.90; 95%CI, 1.76–4.78; *P* < 0.001) were significant predictive factors of a COPD diagnosis (Table 4).

**Table 4:** Significant factors for COPD diagnosis in all subjects

	Univariate analysis			Multivariate analysis		
	OR	95% CI	*P*-value	OR	95% CI	*P*-value
**Age (years)**	**1.11**	**(1.09,1.13)**	**< 0.001**	**1.10**	**(1.07,1.13)**	**< 0.001**
Male	1.02	(0.49,2.13)	0.954			
Height (cm)	0.95	(0.92,0.97)	< 0.001	1.01	(0.97,1.05)	0.786
Weight (kg)	0.93	(0.91,0.95)	< 0.001	0.98	(0.95,1.01)	0.143
Smoking history						
Current smoking	0.92	(0.65,1.3)	0.631			
Pack-years	1.02	(1.01,1.03)	< 0.001	1.01	(1.00,1.02)	0.059
Co-morbidity						
Pulmonary tuberculosis	2.78	(1.79,4.31)	< 0.001	1.17	(0.66,2.10)	0.587
Asthma	13.11	(8.27,20.79)	< 0.001	1.88	(0.97,3.64)	0.060
Lung function test						
**FVC % predicted**	**0.95**	**(0.94,0.97)**	**< 0.001**	**1.03**	**(1.01,1.05)**	**0.006**
**FEV**_**1**_**% predicted**	**0.93**	**(0.92,0.94)**	**< 0.001**	**0.95**	**(0.93,0.96)**	**< 0.001**
FEV_1_/FVC	0.001	(0.001,0.001)	< 0.001			
Self-reported respiratory symptoms						
Cough for more than 3 months	37.80	(15.55,91.87)	< 0.001	2.40	(0.24,24.32)	0.458
Sputum for more than 3 months	17.78	(7.81,40.52)	< 0.001	0.48	(0.02,10.90)	0.647
**Dyspnea**	**25.49**	**(13.68,47.49)**	**< 0.001**	**3.07**	**(1.23,7.68)**	**0.017**
**Wheezing**	**7.83**	**(5.42,11.31)**	**< 0.001**	**2.90**	**(1.76,4.78)**	**< 0.001**
Chronic bronchitis	23.21	(10.67,50.5)	< 0.001	2.76	(0.07,109.05)	0.588

Statistically significant data are presented as bold

*COPD* chronic obstructive pulmonary disease, *FEV*_*1*_ forced expiratory volume for 1 s, *FVC* forced vital capacity, *OR* odds ratio, *CI* confidence interval

### Comparison of baseline characteristics, medical utilization, and costs between PRISm patients with and without medically diagnosed COPD

Among the 316 subjects with PRISm, 16 patients were medically diagnosed with COPD during the 3-year follow-up period. Subjects with medically diagnosed COPD were older, shorter, weighed less, more often had asthma and decreased FVC, and more frequently had dyspnea and wheezing. Due to frequent hospital and medical utilization, their hospitalization cost was greater than that of subjects without medically diagnosed COPD (Table 5).

**Table 5:** Comparison of baseline characteristics, medical utilization, and costs between PRISm with and without medically diagnosed COPD

	PRISm with medically diagnosed COPD	PRISm without medically diagnosed COPD	*P*-value
Age	70.06 ± 7.48	55.21 ± 10.49	< 0.001
Male, n (%)	16 (100.0)	270 (90.9)	–
Height (cm)	162.95 ± 6.9	167.0 ± 6.89	0.023
Weight (kg)	61.58 ± 13.13	69.04 ± 11.41	0.012
Smoking history			
Current smoking, n (%)	8 (50.0)	185 (62.3)	0.325
Pack-years	36.63 ± 14.16	33.02 ± 20.62	0.490
Co-morbidity, n (%)			
Pulmonary tuberculosis	1 (6.3)	20 (6.7)	0.940
Asthma	4 (25.0)	11 (3.7)	< 0.001
Lung function test			
FVC % predicted	64.83 ± 10.86	72.96 ± 9.2	< 0.001
FEV_1_% predicted	69.77 ± 9.16	72.97 ± 6.55	0.188
FEV_1_/FVC	0.76 ± 0.06	0.77 ± 0.06	0.182
Respiratory symptoms, n (%)			
Cough for more than 3 months	0	2 (0.7)	–
Sputum for more than 3 months	0	2 (0.7)	–
Dyspnea	2 (12.5)	1 (0.3)	< 0.001
Wheezing	6 (37.5)	31 (10.4)	0.001
Chronic bronchitis	0	2 (0.7)	–
OPD visit, n (%)	15 (93.8)	7 (2.4)	< 0.001
No. of OPD visit	8.81 ± 10.15	0.03 ± 0.18	< 0.001
Hospitalization, n (%)	9 (56.3)	20 (6.7)	< 0.001
ER visit, n (%)	5 (31.3)	7 (2.4)	< 0.001
ICU admission, n (%)	2 (12.5)	4 (1.4)	0.002
Total hospital visit, n (%)	16 (100.0)	25 (8.4)	–
ICS, n (%)	4 (25.0)	1 (0.3)	< 0.001
ICS + LABA, n (%)	8 (50.0)	3 (1.0)	< 0.001
LAMA, n (%)	4 (25.0)	–	–
SAMA, n (%)	8 (50.0)	4 (1.4)	< 0.001
SABA, n (%)	8 (50.0)	3 (1.0)	< 0.001
Systemic bronchodilator, n (%)	9 (56.3)	2 (0.7)	< 0.001
Methylxanthine, n (%)	11 (68.8)	6 (2.0)	< 0.001
Total prescribed medication, n (%)	16 (100.0)	10 (3.4)	–
Hospitalization medical Cost (for 3 years) (USD)	3647.51 ± 4773.55	223.58 ± 1535.45	0.012
Total	16	297	

Data are presented as mean ± standard deviation or number (percentage)

*PRISm* preserved ratio impaired spirometry, *COPD* chronic obstructive pulmonary disease, *FEV*_*1*_ forced expiratory volume for 1 s, *FVC* forced vital capacity, *OPD* outpatient department, *ER* emergency room, *ICU* intensive care unit, *ICS* inhaled corticosteroid, *LABA* long-acting beta-2 agonist, *LAMA* long-acting muscarine antagonist, *SAMA* short-acting muscarine antagonist, *SABA* short-acting beta-2 agonist

### Significant factors for COPD diagnosis in PRISm

In multivariate analysis of subjects with PRISm, the possibility of COPD diagnosis was increased to 14.0% for every year that subjects aged (OR, 1.14; 95% CI, 1.05–1.24; *P* = 0.002). Wheezing (OR, 4.56; 95% CI, 1.08–19.35; *P* = 0.040) was a significant factor for a diagnosis of COPD in PRISm patients (Table 6).

**Table 6:** Significant factors for COPD diagnosis in PRISm

	Univariate analysis			Multivariate analysis		
	OR	95% CI	*P*-value	OR	95% CI	*P*-value
**Age (years)**	**1.14**	**(1.08, 1.21)**	**< 0.001**	**1.14**	**(1.05, 1.24)**	**0.002**
Male						
Height (cm)	0.93	(0.87, 0.99)	0.025	1.03	(0.92, 1.16)	0.564
Weight (kg)	0.94	(0.9, 0.99)	0.013	0.95	(0.89, 1.02)	0.153
Smoking history						
Current smoking	0.61	(0.22, 1.66)	0.329			
Pack-years	1.01	(0.99, 1.03)	0.490			
Co-morbidity						
Pulmonary tuberculosis	0.92	(0.12, 7.35)	0.940			
Asthma	8.67	(2.41, 31.23)	0.001	5.87	(0.94, 36.56)	0.058
Lung function test						
FVC % predicted	0.93	(0.89, 0.97)	0.001	1.01	(0.95, 1.09)	0.694
FEV_1_% predicted	0.95	(0.9, 1.01)	0.071			
FEV_1_/FVC	0.001	(0.001, 35.7)	0.183			
Self-reported respiratory symptoms						
Cough for more than 3 months						
Sputum for more than 3 months						
Dyspnea	42.29	(3.61, 494.74)	0.003	8.88	(0.65, 121.7)	0.102
**Wheezing**	**5.15**	**(1.75, 15.14)**	**0.003**	**4.56**	**(1.08, 19.35)**	**0.040**
Chronic bronchitis						

Statistically significant data are presented as bold

*COPD* chronic obstructive pulmonary disease, *PRISm* preserved ratio impaired spirometry, *FEV*_*1*_ forced expiratory volume for 1 s, *FVC* forced vital capacity, *OR* odds ratio, *CI* confidence interval

## Discussion

We investigated the incidence of COPD in PRISm patients and sought to identify significant risk factors of COPD in PRISm patients. We found that PRISm patients were 4 times more likely to receive a COPD diagnosis than a normal group. Sood et al. have also reported a high COPD incidence in PRISm patients (about double that in the normal population) [24]. We also showed that PRISm patients paid more hospital visits, used more prescribed COPD medications, and accounted for an increased economic burden. Despite not meeting COPD criteria, these patients require careful observation because of their risk for COPD development and concomitant medical utilization. PRISm occurs in about 6.6–17.6% of the general global population [15, 25, 26]; nevertheless, PRISm remains poorly understood. Many clinicians miss this “unclassified” or “non-specific” group, and discharge them without explanation, warning, or follow-up appointment. Detecting and treating these early-stage patients is requisite.

Some subjects with PRISm might have underlying restrictive lung disease. Significantly lower FVC (72.55 ± 9.45%) in PRISm patients than in normal (92.96 ± 10.02; *P* < 0.001) and unrevealed COPD (88.51 ± 15.02; *P* < 0.001) subjects supports this supposition. However, Wan et al. reported that a true restrictive pattern, defined by total lung capacity, was not frequently observed in PRISm [10]. This should be elucidated in further studies.

Subjects in the PRISm group had a heavier smoking history, more severe respiratory symptoms and decreased lung function, and more frequent co-morbidity than the normal population; these differences were less marked when compared to the unrevealed COPD group. However, we found that the prevalence of current smoking in the PRISm group was higher than that in both the normal and unrevealed COPD groups. It may be that many current-smokers in the PRISm group did not experience respiratory symptoms, did not visit hospitals, and were not warned to stop smoking. Current-smokers in the PRISm group may develop COPD unless they stop smoking, as previously shown [24]. Doctors should check the smoking status in PRISm patients more carefully, and should strongly recommend that they stop smoking.

Although age, lung function, dyspnea, and wheezing are significant predictive factors of COPD in the subjects overall, only age and wheezing were significant predictive factors for a COPD diagnosis in PRISm patients. Both age [27] and wheezing [28] are well-known predictive factors for COPD.

Lung function was not a significant predictive factor of COPD in PRISm. Low FEV_1_ was a significant predictive factor of COPD overall, but not in PRISm patients specifically. The preserved ratio which is shown in PRISm means that these patients rarely have an extremely reduced FEV_1_. In fact, Table 1 shows a relatively small standard deviation of FEV_1_ in PRISm patients, as compared to other groups, although the number of subjects was small. It implies FEV_1_ in PRISm has small predictive power for prognosis. Thus, it is necessary to monitor PRISm subjects carefully, even in the absence of severe reduced FEV_1_.

Additionally, relatively preserved FVC was a significant predictive factor for COPD in the overall cohort using multivariate analysis, but not in PRISm patients. The reasons why preserved FVC is significant risk factor for COPD are as follows. Before adjustment, FVC in subjects with medically diagnosed COPD (81.14 ± 15.77%) was significantly lower than that in subjects without COPD (89.85 ± 12.85%; *P* < 0.001). We can easily assume that preserved FVC will be protective factor for COPD, however results were contrary to that in multivariate analysis with adjustment. This indicates that other associated co-variables affected the findings of FVC in multivariate analysis. We speculated FEV_1_ might be contributing factor for this confusing result. The decline in FEV_1_ was much larger than that in FVC in Table 3, and FVC is unavoidably influenced by changes in FEV_1_. Therefore, we speculated that FEV_1_, as a co-variable, might have affected the FVC findings in multivariate analysis with adjustment.

Unrevealed COPD implies a significantly impaired FEV_1_/FVC ratio, meeting the standard COPD spirometry criteria for airway obstruction, but without a clinical diagnosis of COPD, no hospital visits, and no use of COPD medication to date. The number of subjects with unrevealed COPD was double that of the PRISm group in this study. Coultas et al. showed a similar proportion of undiagnosed COPD (79.7%) in the USA [3]. Chung et al. have shown that, in Korea, 97% of COPD cases are undiagnosed [2], or misdiagnosed [29]; their diagnosis and treatment should be addressed, because unrevealed COPD also leads to more hospital visits, increased medication use, and an increased economic burden [30].

Woodruff et al. showed that smokers with normal lung function commonly experience respiratory symptoms and exacerbations. They suggested a new entity that includes smoking-related chronic pulmonary disease [6]. Other recent studies also suggest that the pre-COPD stage is clinically and medically important [31, 32]. We assume that PRISm may also be a pre-COPD-stage chronic pulmonary disease. PRISm patients should be advised to have regular check-ups to monitor COPD development, and more so if they have advance aged or wheezing, irrespective of the severity of lung function decrease (FEV_1_).

This study had some limitations. First, “medically diagnosed COPD” may be considered artificial. “COPD incidence” is not an accurate term, but in this study reflects the incidence of medically diagnosed COPD as defined by the HIRA data, which includes insurance claims but not pulmonary function test data. However, the previously reported COPD incidence (2.6–9.2/1000 PY) [27, 33–35] is not markedly different from that in this study (4.4/1000 PY in normal; 17.0/1000 PY in PRISm). “Medically diagnosed COPD” with hospital visits and medication use is more relevant than COPD diagnosed based only on impaired lung function (FEV_1_/FVC < 0.7), without medical utilization. Therefore, this artificial definition may be appropriate for use in this study. Second, this cohort study did not include follow-up pulmonary function tests, because the KNHANES conducted pulmonary function tests in different populations each year.

## Conclusions

PRISm is likely to develop into COPD over time, and it leads to frequent hospital visits, increased medication use, and greater hospitalization costs. Subjects with PRISm should be carefully monitored for COPD development, especially when they are older or have wheezing, regardless of lung function.

## Abbreviations

CI: confidence interval
COPD: chronic obstructive pulmonary disease
FEV_1_: forced expiratory volume for 1 s
FVC: forced vital capacity
OR: odds ratio
PRISm: preserved ratio impaired spirometry
PY: pearson-year
